# Maternal and neonatal viromes indicate the risk of offspring's gastrointestinal tract exposure to pathogenic viruses of vaginal origin during delivery

**DOI:** 10.1002/mlf2.12034

**Published:** 2022-08-25

**Authors:** Jinfeng Wang, Liwen Xiao, Baichuan Xiao, Bing Zhang, Zhenqiang Zuo, Peifeng Ji, Jiayong Zheng, Xiaoqing Li, Fangqing Zhao

**Affiliations:** ^1^ College of Food Science and Nutritional Engineering China Agricultural University Beijing China; ^2^ Beijing Institutes of Life Science Chinese Academy of Sciences Beijing China; ^3^ University of Chinese Academy of Sciences Beijing China; ^4^ Department of Gynecology and Obstetrics Wenzhou People's Hospital Wenzhou China

**Keywords:** bacteriophage, gastrointestinal tract, newborn, virus

## Abstract

A cumulative effect of enterovirus and gluten intake on the risk of celiac disease autoimmunity in infants highlights the significance of viral exposure in early life on the health of children. However, pathogenic viruses may be transmitted to the offspring in an earlier period, raising the possibility that women whose vaginas are inhabited by such viruses may have had their babies infected as early as the time of delivery. A high‐resolution intergenerational virome atlas was obtained by metagenomic sequencing and virome analysis on 486 samples from six body sites of 99 mother–neonate pairs. We found that neonates had less diverse oral and enteric viruses than mothers. Vaginally delivered newborns seconds after birth had a more similar oral virome and more viruses of vaginal origin than cesarean‐section (C‐section) newborns (56.9% vs. 5.8%). Such viruses include both *Lactobacillus* phage and potentially pathogenic viruses, such as herpesvirus, vaccinia virus, and hepacivirus, illustrating a relatively high variety of the pioneer viral taxa at the time of delivery and a delivery‐dependent mother‐to‐neonate transmission along the vaginal–oral–intestinal route. Neonates are exposed to vaginal viruses as they pass through the reproductive tract, and viruses of vaginal origin may threaten their health. These findings challenge the conventional notion that vaginal delivery is definitely better than cesarean delivery from the perspective of microbial transmission. Screening for vaginal virome before delivery is a worthwhile step to advocate in normal labor to eliminate the risk of intergenerational transmission of pathogenic viruses to offspring.

## Impact statement

Both gut microbiome and virome of early life have always drawn great attention because they are strongly associated with the health status of an individual in the future. This study presents a high‐resolution maternal–infant intergenerational virome atlas with the largest population size and body sites to date and elucidates the impact of different delivery modes on shaping neonatal virome in which most of the viruses, including herpesvirus and poxvirus, detected in the neonatal oral cavity and gut can be traced back to the maternal vagina during vaginal delivery. This study to some extent challenges the conventional notion that vaginal delivery is definitely better than cesarean delivery through in‐depth analysis of virome data from a large mother–infant paired population.

## BACKGROUND

In the early years of life, despite the scarcity of microbial species and numbers in the newborn, the role microbes play cannot be ignored^1^, ^2^. A large number of studies have indicated that the acquisition, colonization, and succession of early‐life gut microbiota form a mucosal barrier, on the one hand, helping to resist some pathogenic bacteria and providing protection to newborns with immature immune function^3^, ^4^, ^5^; on the other hand, gut microbiota stimulates the development of the digestive, metabolic, immune, and nervous systems of infants and children, which is essential to promote the digestion and absorption of nutrients, maintain intestinal immune homeostasis and reduce allergic reactions^6^. Along with bacteria^7^, viruses also play a part in these functions.

The gut virome, consisting of phages and eukaryotic viruses, directly or indirectly affects infant and child health. As the dominant component in gut virome, phages shape the early life microbiota by preying on bacteria. In a previous study, the infant gut phage–bacteria relationship during the first 2 years of life was consistent with the classical Lotka–Volterra “predator–prey” model, accompanied by phage community contraction and expansion of bacterial richness and diversity^8^. Our earlier analysis of more than 10,000 infants aged 0–3 years also suggests that, in early life, phages may participate in the dynamic regulation of gut microbiota and deterministic shifts of enterotype^9^. Phages not only target their host bacteria but may also spread to nonhost bacteria via interactions, resulting in a wide range of effects on the entire gut microbiota and its metabolism, such as changing the concentration of neurotransmitters^10^. Besides phages, eukaryotic viruses can be detected in early life^8^. These eukaryotic viruses are more likely to have a direct effect on infants than phages. In infants of 1–2 years old, a cumulative effect of enterovirus and gluten intake on the risk of celiac disease autoimmunity was observed^11^. Considering their close association with infant and child health, the origin of phages and viruses has attracted attention.

Mothers are widely recognized as one of the major sources of early‐life microbes, including phages and eukaryotic viruses. The migratory routes of several notorious pathogenic eukaryotic viruses are well defined. An example from the list is the Zika virus, whose epidemic history began in 2007 and emerged in Brazil in 2015, infects pregnant women, and causes microcephaly and severe neurological complications in the fetus or newborn through maternal–fetal transmission^12^. In contrast, little is known about when and how maternal phages and viruses are transmitted to offspring in the noninfected status. To improve the understanding of intergenerational transmission, some studies in recent years investigated the virome of infants and their mothers and found that viruses may be transmitted to the offspring during pregnancy and postpartum^13^, ^14^. Such transmission can be influenced by a variety of factors. For example, neonatal enterovirus diversity and detection rates vary with maternal blood glucose levels^15^, whereas after birth breast milk confers infant bifidophages^16^. A few studies focused on the effect of delivery mode on the infant virome, suggesting that infants born by cesarean‐section (C‐section) may have a different intestinal viral diversity and phage–bacterial interactions than infants born vaginally due to the absence of corresponding viruses from the vagina^17^, ^18^. However, by far, no studies report the neonatal virome at the moment of birth, and there is a lack of knowledge of the first, true “pioneer” viruses at the time of delivery.

In this study, we collected oral secretions from more than 90 newborns seconds after birth, together with paired meconium and samples from multiple body sites of their mothers, and performed whole‐genome shotgun (WGS) sequencing and virome analysis to explore the origin and accumulation of human initial phages and eukaryotic viruses, focusing on the transmission of pathogenic eukaryotic viruses between mothers and newborns during delivery. According to the findings, we propose here a special initiative that women with such viruses residing vaginally may be alerted to the viral transmission to their children when choosing vaginal delivery.

## RESULTS

### Highly diverse viruses reside in neonates

**Figure 1:**
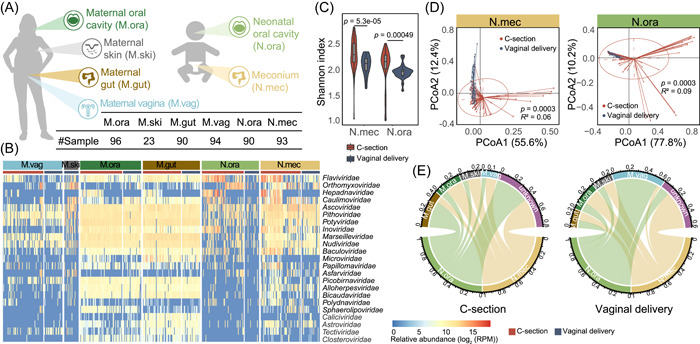
Sample collection and virome comparison between C‐section and vaginally delivered neonates and their mothers. (A) Samples were collected from six body sites, including oral (M.ora), intestinal (M.gut), vaginal (M.vag) and skin (M.ski) of mothers, and oral (N.ora, oral contents collected within seconds after birth) and intestinal (N.mec, meconium, i.e., the first excretion after birth) of newborns. The number of each sample type is listed in the table. (B) Relative abundance of viruses in each body site is profiled at the family level. The red and blue bars represent samples of subjects delivered with C‐section and vaginal mode, respectively. The virus families with the largest intergroup variance (the intergroup variance of abundance is greater than the overall median variance) are shown. Since no skin samples of C‐section mothers were collected, only the M.ski of vaginal delivery is used here. (C) Alpha diversities of viruses in N.mec (left) and N.ora (right) were calculated at the family level and are colored by delivery modes. Violins show median (black horizontal line), quartiles (edge of boxes), and kernel density estimation (violin) for each distribution. (D) Principal co‐ordinates analysis (PCoA) of virome profiles in N.mec (left) and N.ora (right) was performed at the family level. (E) Viral source tracking from mothers to neonates delivered by C‐section (left) and vagina (right), respectively. The proportion of shared viruses between generations was measured at the species level.

In this study, we recruited 99 mother–newborn pairs (32 vaginal delivery and 67 C‐section) and collected 486 samples from 6 body sites, including saliva (M.ora), stool (M.gut), vagina (M.vag), and skin (M.ski) of mothers, and oral contents (N.ora) and the first excretion (meconium, N.mec) of newborns (Figure 1A), and eventually generated a total of 3.85 Tb metagenomic sequencing data to identify viruses.

Based on this large scale of metagenomic data, we performed virome analysis and found that neonatal pioneer viruses were exceedingly luxuriant in taxonomy, including the predominant taxa, *Siphoviridae* and *Myoviridae* (Figure S1), and were variable in relative abundance across generations and body sites, with half (23 out of 46) of the detected viral families varying greatly (Figure 1B). We also found that the viruses residing in meconium were particularly abundant compared to those in the neonatal oral cavity and in the maternal skin and vagina, indicating an extensive accumulation of viruses inside the gastrointestinal (GI) tract at birth.

### Neonatal virome varies by mode of delivery

To track the source of the neonatal viruses, in addition to inspecting the viral transmission between mothers and offspring during delivery, we compared the virome of neonates born with different delivery modes. Shannon index was calculated and showed that the viral diversity was significantly lower in vaginally delivered newborns than in those born by C‐section (Wilcoxon's rank‐sum test, *p* < 0.05) (Figure 1C). Alpha diversities calculated with different indices at three taxonomic levels resembled each other (Figure S2A).

We then performed the principal co‐ordinates analysis (PCoA) to assess the effect of delivery modes on the structure of the viral community (Figures 1D and S2B), and observed the separation of the neonatal samples into two distinct clusters (Adonis test, *p* < 0.05) and a more similar virome profile among vaginally delivered newborns, which may be attributed to a uniform maternal viral transmission while passing through the birth canal. This hypothesis was confirmed by tracking the source of viruses in neonates (Figure 1E): C‐section neonates had about 40% of viruses of unknown origin, whereas vaginally delivered neonates acquired a large number of viruses from the maternal vagina; the proportion of viruses of vaginal origin in the neonatal oral cavity was particularly high (56.9%), presumably with direct oral exposure to the vagina that allowed vaginal viruses to instantly colonize during natural delivery.

### Vaginally delivered neonates harbor more vaginal phages

**Figure 2:**
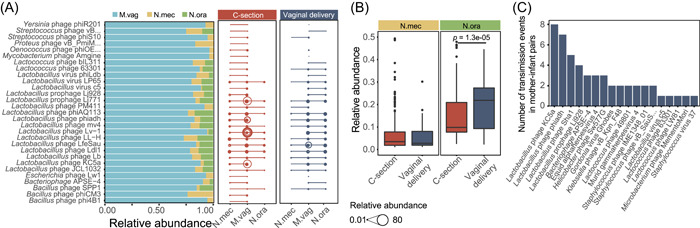
Phage transmission from mothers to offspring evaluated based on metagenomic reads. (A) Relative abundance of vagina‐enriched phages in three body sites (M.vag, N.mec, and N.ora) was counted. Left panel shows the vagina‐enriched phages whose proportions in the vagina are greater than 70%, and the right panel shows transmission events of these phages between mothers and offspring. The size of bubbles represents the relative abundance of phages. (B) Relative abundance of the vagina‐enriched phages transmitted from mother's vagina to neonate. Box plots show median (black horizontal line), 25th and 75th quartiles (edge of boxes), and upper and lower extremes (whiskers). (C) Transmission events of phages in vaginally delivered mother–infant pairs.

To measure the transmission scale of vaginal‐bearing viruses to offspring, we counted shared phages between newborns and vaginal tracts. The results showed that the oral cavity of vaginally delivered neonates harbored more vaginal phages (Wilcoxon's rank‐sum test, *p* < 0.05) than that of C‐section neonates and that of the meconium of vaginally delivered neonates (Figure 2A,B), confirming a fast spread of viruses along the vaginal–oral route at birth. We then tracked the transmission events in sequencing reads of paired maternal and neonatal samples and found that a large number of those shared vaginal phages were *Lactobacillus* phages (Figure 2C).

We next assembled reads into contigs, annotated and identified phage contigs, and counted the number of the neonatal reads mapping to each phage contig of vaginal origin. The finding of significantly more frequent transmissions detected in the reads‐based analysis was reproducible in the contig‐based analysis (Wilcoxon's rank‐sum test, *p* < 0.05) (Figure S3A). Likewise, when measuring the transmission of vaginal phages in contigs of mother–infant pairs, the ratio was the highest in the oral cavity of vaginally delivered neonates (Figure S3B,C), and transmission events were more likely to occur in the predominant taxa, *Siphoviridae* and *Myoviridae* (Figure S3D).

### Natural birth increases the risk of virus transmission

**Figure 3:**
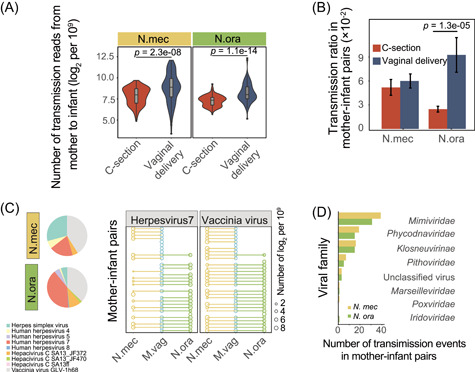
Transmission of eukaryotic viruses from the maternal vagina to neonatal meconium or oral cavity. (A) Number of metagenomic reads of neonatal meconium (N.mec) and oral cavity (N.ora) samples that could be mapped to the eukaryotic virus contigs of the maternal vagina, respectively. The read count is normalized to 10^9^ and log_2_‐transformed. Violins show median (black horizontal line), quartiles (gray box), and kernel density estimation (violin) for each distribution. (B) Transmission ratio of eukaryotic virus contigs in mother–infant pairs. Error bars indicate the standard deviation. (C) Viral composition of vaginally delivered neonates (left panel) and transmission patterns from the vagina in each vaginally delivered mother–infant pair (right panel). The two most prevalent viruses are shown as examples. The size of bubbles represents the relative abundance of viruses. (D) Transmission events of other viruses in vaginally delivered mother–infant pairs.

To assess the prospective risks of vaginally carried viruses to newborns by vaginal delivery, we specifically focused on eukaryotic viruses shared between mother and neonate. According to the number of metagenomic reads from neonatal samples that could be mapped to eukaryotic virus contigs from the maternal vagina, vaginally delivered newborns acquired a significantly larger (Wilcoxon's rank‐sum test, *p* < 0.05) number of vaginal viruses than C‐section newborns (Figure 3A). A similar result was observed by analyzing virome data from mother–infant pairs (Figures 3B and S4A–B), which suggests a frequent transmission of the eukaryotic virus during vaginal delivery.

**Figure 4:**
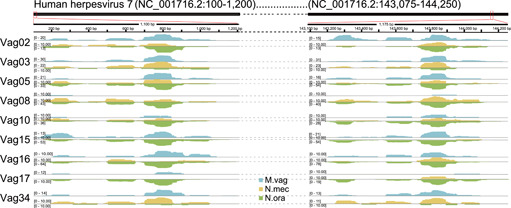
Metagenomic reads of mothers and their offspring mapped to a human herpesvirus genome. Three sample types, including vaginal (M.vag) of the mother, and oral (N.ora) and intestinal (N.mec) of the newborn from nine mother–infant pairs, were used. Sample types were assigned to different colors. The genome of one strain of human herpesvirus 7 (NC_001716.2) was selected as the reference. The number on the left represents the sequencing depth of each sample. All samples used are from neonates delivered vaginally and their mothers.

For the viruses of neonates vaginally delivered and transmission events from the vagina in each vaginally delivered mother–infant pair, *Mimiviridae*, *Orpheovirus*, and *Megavirus* had the highest detection rates, and other pathogenic viruses, such as herpesvirus, vaccinia virus, and hepacivirus, were also found to be capable of frequently inoculating into the GI tract of the newborn during vaginal delivery with high phylogenetic diversities (Figures 3C,D and S4C,D). The existence of these viruses in vaginal, neonatal oral, and meconium samples and co‐occurrence of mother‐to‐child transmission associated with vaginal delivery were further confirmed by the evenly mapped reads from those samples to the viral reference genomes (Figures 4 and S5).

## DISCUSSION

Although the importance of the virome in the establishment of the gut microbiota has gradually been recognized^14^, more information is needed to understand the origin and factors influencing the neonatal virome. In this study, we obtained neonatal fecal samples from nearly 100 deliveries at the start of life, and together with paired samples from multiple body sites of their mothers, we explored the landscape of the earliest viral residents in the human body and unveiled an unprecedented diversity of viral taxa from newborns. By tracing the events of eukaryotic virus transmission across generations, we revealed more clearly the relationship between the acquisition of the human virome and the delivery mode.

Recently, there has been some controversy regarding the diversity of eukaryotic viruses in infants in early life. Previous studies have pointed to a high diversity of viruses in infant feces, including RNA viruses (e.g., enterovirus, parechovirus, tombamovirus) and DNA viruses (e.g., anellovirus)^8^, ^18^. A recent study, however, reported that virus‐like particles are largely absent from fetal and early fecal samples and it is not until about 4 months of life that lytic phages and eukaryotic viruses begin to appear in the gut^19^. It should be noted that all these prior studies used stool samples from hours, days or even months after birth, which cannot fully represent the acquisition of viruses in the earliest stages of life. In this study, we used much earlier neonatal samples, from which a large number of viral sequences, both phage and eukaryotic, were detected. In particular, the oral samples were taken from newborns a few seconds after delivery, which represents the initial state of the neonatal virome, suggesting that viruses have been there at birth and are not largely absent in newborns as previously thought. These results also demonstrate that some viruses may have been present before birth through maternal–fetal transmission, as in the case of the Zika virus.

Since the mother is the most important source of the initial microbes in the offspring's GI tract^20^, ^21^, the prevalence of viruses that newborns acquire from their mothers may, like bacteria^22^, vary depending on the delivery mode and, in turn, on the health of the offspring. Several recent studies explored the relationship between infant virome and the mode of delivery and found that in the first year of age, infants vaginally delivered had higher enterovirus diversity than those delivered by cesarean section^17^, ^18^. We found here that the abundance of viruses like *Flaviviridae*, *Orthomyxoviridae*, and *Hepadnaviridae* was higher in C‐section neonates than in vaginally delivered neonates, which might increase the risk of infection and alert to the interfusion of harmful viruses of environmental origin. We also showed that vaginal delivery conferred more *Lactobacillus* phage in the offspring compared to C‐section delivery. One possible reason for this may be the predominance of *Lactobacillus* in the vaginal microbiota of pregnant women^23^, ^24^, ^25^, and *Lactobacillus* phage from the mother, together with host bacteria, may participate in the regulation of neonatal gut microbiota. The fact that *Lactobacillus* seldom becomes the dominant species in the gut of newborns^9^, may be due to indirect regulation of such numerous, vaginally acquired *Lactobacillus* phages.

Many studies have attempted to examine the relationship between delivery mode and the rate of mother‐to‐child transmission of human immunodeficiency virus, hepatitis B virus and herpes simplex virus, and have provided preliminary evidence that C‐section reduces the risk of transmission of these viruses compared to vaginal delivery^26^, ^27^, ^28^, ^29^. We set inclusion and exclusion criteria, such as maternal diseases, gestational age, diet and medication use to adjust the effect of host factors, that may affect human virome. A high frequency of herpesvirus, vaccinia virus, and *Mimiviridae* in vaginally delivered neonates and their mothers was detected in a large population, which may pose a risk of respiratory infection and inflammation^30^. This is a reminder to be aware of the transmission of diverse eukaryotic viruses between mother and child associated with vaginal delivery.

Our study indicates that newborns are exposed to vaginal viruses as they pass through the reproductive tract, which may have potential threats to their health. Thus, the benefits of natural labor are not absolute but have prerequisites from the perspective of viral transmission. The new revelation that these findings bring to reproductive health is that a recommendable procedure for normal labor may include screening the vaginal virome before delivery and being alert to the risk of intergenerational transmission of pathogenic viruses to the offspring's GI tract.

## MATERIALS AND METHODS

### Study cohorts and sample collection

A group of volunteers including newborns and their mothers were recruited at Wenzhou People's Hospital. These volunteers were all Han Chinese, local permanent residents of Wenzhou, China. The included mothers were nonvegetarians, did not use any antibiotic‐based or hormonal drugs during pregnancy, had no history of smoking or alcohol abuse, and were without systemic or metabolic diseases. The included newborns were all defect‐free and born at term.

After obtaining informed consent from the mother and the parents or guardians of the newborn, we collected six sample types, including M.ora, M.gut, M.vag, and M.ski swabs from mothers before delivery, and N.ora and the N.mec from newborns at birth or after delivery using sterile tools and containers as described previously^15^, ^31^. All samples were stored in sterile containers and frozen at −80°C in the freezer.

### Metagenomic sequencing and data processing

Metagenomic DNA was extracted from all samples using the QIAamp DNA Mini Kit (Qiagen), and the resulting DNA was used for WGS sequencing. Briefly, DNA was sheared into fragments of approximately 300 bp in length using an ultrasonicator (Covaris). Subsequently, random‐fragment libraries were constructed using the Nextera DNA Sample Preparation Kit (Illumina) according to the instructions. After qualification and quantification of the libraries by Fragment Analyzer (Advanced Analytical Technologies) and quantitative PCR, the libraries were finally sequenced on the HiSeq 2500 platform (Illumina) for 150‐bp paired‐end sequencing. All these operations were conducted using sterile tools in a strictly controlled and sterile workplace. DNA‐free water was used as a negative control to rule out possible contaminations from environmental or reagent sources.

Raw sequencing data were filtered using FASTQC and removed adapter and barcode with Trimmomatic (v0.32)^32^. High‐quality reads were aligned to the hg38 version of the human reference genome using the Burrows‐Wheeler Alignment Tool (v0.7.17) software^33^ with default parameters to remove human‐derived sequences. If one of the paired‐end reads matched the hg38 human genome, the entire read pair was discarded.

### Virus identification from reads

Using DIAMOND (v0.9.31.132)^34^, the filtered reads were blasted against the NR database with the cutoff of e value 1e − 5. The viral reads were annotated in the result file with reference to the “taxonomy ID ~ scientific name” table published by NCBI (https://ftp.ncbi.nih.gov/pub/taxonomy/). The relative abundance of viruses classified in each sample was finally obtained by counting the number of hits to each virus.

### Assembly, gene prediction, and annotation

High‐quality reads were assembled into contigs with the parameter of –k‐min 15 –k‐max 141 –k‐step 2 in MEGAHIT (v1.2.9)^35^, the parameter of –k $klist ‐meta in metaSPAdes (SPAdes‐3.14.1)^36^, and the parameter of –mink 24 –maxk 120 –step 4–min_contig 200 in IDBA_UD (version 2)^37^. All contigs obtained from these three assembly tools were integrated, and the redundancy removal was performed with the parameter of cd‐hit‐est ‐i $input ‐o $output ‐d 0 ‐M 10000 ‐T 16 in CD‐HIT (version 4.6)^38^ to obtain the unique contigs as the final assembly result.

For the viral contigs with a length of at least 3000 bp, we used the default parameters of Prodigal (v2.6.3)^39^ and selected the “meta” mode to predict the open‐reading frames (ORFs). Then, the predicted ORFs were aligned to viral sequences from RefSeq using BLAST (v2.8.1)^40^, and best hits were chosen as the taxonomic results. A contig was annotated to a viral species if two thirds or more of the annotated ORFs in all ORFs of this contig were assigned to the same viral species.

### Abundance calculation for viral contigs

The reads of each sample were mapped to the annotated viral contigs and the genomes of human susceptible viruses, including adenovirus, herpesvirus, poxvirus, and hepatitis virus using Bowtie 2^41^, and the number of reads of each viral contig and genome was calculated for each sample. Normalization was performed by dividing the number of reads mapped to the contig by the total number of reads contained in each sample. To facilitate visualization, the read count of each sample was normalized to 10^9^ and log_2_‐transformed.

### Viral transmission rate estimation

The mother–infant viral transmission rate was calculated by taking all samples with a normalized value greater than 0.1. The transmission rate was interpreted as the proportion of each viral contig co‐occurring between all mother–infant pairs. For each mother–infant pair with cesarean or vaginal delivery, the number of times the contig appeared in both the infant (meconium or oral cavity) and the mother's vaginal samples was counted, and then the number of times the contig appeared in the infant or the mother's vaginal samples was counted, and the ratio of the two was defined as the transmission rate of the viral contig.

## AUTHOR CONTRIBUTIONS

Fangqing Zhao conceived the study. Fangqing Zhao and Jinfeng Wang designed the study and prepared the manuscript. Zhenqiang Zuo, Jiayong Zheng and Xiaoqing Li collected the samples and conducted the experiments. Jinfeng Wang, Liwen Xiao, Baichuan Xiao, Bing Zhang, Peifeng Ji, and Fangqing Zhao analyzed the data. All authors approved the final version of the manuscript.

## ETHICS STATEMENT

This study was approved by the Medical Ethics Committee of Wenzhou People's Hospital (No. 2019‐099). Informed consent was obtained from the mother and the parents or guardians of the newborn.

## CONFLICT OF INTERESTS

The authors declare no conflict of interests.

## Supporting information

Supporting information.

## Data Availability

Raw metagenomic sequencing data have been deposited in the Sequence Read Archive database under accession number PRJNA695070.
