# Supplementary material for: Maternal and neonatal viromes indicate the risk of offspring's gastrointestinal tract exposure to pathogenic viruses of vaginal origin during delivery
Source: mLife. 2022 Aug 25;1(3):303–10. doi: 10.1002/mlf2.12034 (PMC10989755; doi:10.1002/mlf2.12034)
Supplement: Supplementary file 1 — Supporting information. [file MLF2-1-303-s001.docx]

**Figure S1. Viral composition of six maternal and neonatal body sites.** Viromes of six body sites including oral (M.ora), intestinal (M.gut), vaginal (M.vag) and skin (M.ski) of the mother, and oral (N.ora, oral contents collected within seconds after birth) and intestinal (N.mec, meconium, i.e., the first excretion after birth) of the newborn are shown. Taxonomic classification was performed at the family level. The reads count of each sample was normalized to 10^9^ and log2-transformed.

**Figure S2. Viral diversities in neonatal meconium and oral cavity at different taxonomic levels.** (A) Alpha diversities of viruses in neonates at three taxonomic levels, represented as Shannon index (top) and Chao 1 (bottom), respectively. Box plots represent median (black horizontal line), 25th and 75th quartiles (edge of boxes), upper and lower extremes (whiskers). (B) PCoA of viruses in neonates at the genus level (left) and species level (right), respectively.

**Figure S3. Evaluation of phage transmission from mothers to offspring based on contigs.** (A) Number of the reads of neonatal meconium (N.mec) and oral cavity (N.ora) mapping to the phage contigs of maternal vagina, respectively. The reads count was normalized to 10^9^ and log2-transformed. Box plots represent median (black horizontal line), 25th and 75th quartiles (edge of boxes), upper and lower extremes (whiskers). (B) Transmission ratio of phage contigs in mother-infant pairs. Bar plots represent the mean and error bars indicate the standard error. (C) Transmission events of phage contigs between mothers’ vagina and neonates. Only phages transmitted in vaginally delivered infants but not in cesarean infants are shown. The size of bubbles corresponds to the abundance of phages. (D) Transmission events of each phage contig in vaginally delivered mother-neonate pairs.

**Figure S4. Transmission of eukaryotic virus from mothers to offspring evaluated from contigs.** (A) Transmission events of eukaryotic viral contigs between mothers’ vagina and neonates. Only eukaryotic virus contigs transmitted in vaginally delivered but not in cesarean neonates are shown. The size of bubbles corresponds to the abundance of eukaryotic viruses. (B) Transmission reads mapped to each of above eukaryotic virus contigs in mothers-neonate pairs. (C) Transmission events of eukaryotic viral contigs in vaginally delivered mother-neonate pairs. OSN represents Only Syngen Nebraska virus. (D) Phylogenetic tree of eukaryotic viral contigs (red) and the corresponding reference genomes (black). ACE corresponds to Anomala cuprea entomopoxvirus. The number in the phylogenetic tree represents the bootstrapping values, where only those greater than 70 are shown.

**Figure S5. Another example of viral transmission from maternal vagina to neonatal oral cavity.** Reads mapping of six mother-neonate pairs to the viral reference genome, colored by each body site. One genome of Megaviricetes (NC_036594.1) was used as the reference. The number on the left represents the reads coverage of each sample.
